# Nucleated Polymerisation in the Presence of Pre-Formed Seed Filaments

**DOI:** 10.3390/ijms12095844

**Published:** 2011-09-09

**Authors:** Samuel I. A. Cohen, Michele Vendruscolo, Christopher M. Dobson, Tuomas P. J. Knowles

**Affiliations:** Department of Chemistry, University of Cambridge, Lensfield Road, Cambridge CB2 1EW, UK

**Keywords:** protein, aggregation, amyloid

## Abstract

We revisit the classical problem of nucleated polymerisation and derive a range of exact results describing polymerisation in systems intermediate between the well-known limiting cases of a reaction starting from purely soluble material and for a reaction where no new growth nuclei are formed.

## 1. Introduction

The classical theory of nucleated polymerisation [1] describes the growth of filamentous structures formed through homogeneous (primary) nucleation [2–7]. This framework was initially developed by Oosawa and coworkers in the 1960s [1,8] to describe the formation of biofilaments, including actin and tubulin. This theory has been generalised to include heterogeneous (secondary) nucleation processes, such as surface-catalysed nucleation, by Eaton and Ferrone [9–11] in the context of their pioneering work elucidating the polymerisation of sickle haemoglobin, and by Wegner [12,13] in order to include fragmentation processes into the growth model for actin filaments; these processes differ from primary nucleation in that they produce new aggregates at a rate that is dependent on the aggregate concentration, whereas the rate of primary nucleation is dependent on only the concentration of monomeric peptide.

For irreversible growth in the absence of pre-formed seed material and secondary nucleation pathways, in 1962 Oosawa presented solutions to the kinetic equations, which were very successful in describing a variety of characteristics of the polymerisation of actin and tubulin [8]. The other limiting case, namely where seed material is added at the beginning of the reaction and where no new growth nuclei are formed during the reaction, is also well-known and results in a rate law given by a single exponential that is characteristic of first order kinetics. In this paper, we present exact results for filament systems proliferating through primary nucleation which encompass all cases between these limiting scenarios, extending the results of Oosawa for a system dominated by primary nucleation to the case where an arbitrary concentration of pre-formed seed material is present. We also discuss a range of general closed form results from the Oosawa theory for the behaviour of a system of biofilaments growing through primary nucleation and elongation. We then compare the behaviour of systems dominated by primary nucleation to results derived recently for systems dominated by secondary nucleation.

## 2. Results and Discussion

### 2.1. Derivation of the Rate Laws for the Polymer Number and Mass Concentrations

The theoretical description of the polymerisation of proteins such as actin and tubulin to yield functional biostructures was considered in the 1960s [8]. For a system that evolves through primary nucleation of new filaments, elongation of existing filaments, and depolymerisation from the filament ends, the change in concentration of filaments of size *j*, denoted *f*(*j; t*), is given by the master equation [1,8]:

(1) $$\begin{array}{l} \frac{\partial f(t,j)}{\partial t}=2m(t)k_{+}f(t,j-1)-2m(t)k_{+}f(t,j)\\ +2k_{\text{off}}f(t,j+1)-2k_{\text{off}}f(t,j)\\ +k_{n}m{\left(t\right)}^{n_{c}}\delta_{j,n_{c}} \end{array}$$

where *k*_+_, *k*_off_, *k_n_* are rate constants describing the elongation, depolymerisation and nucleation steps and *m*(*t*) is the concentration of free monomeric protein in solution. The factor of 2 in Equation (1) originates from the assumption of growth from both ends. The first two terms in Equation (1) describe the change in the population of filaments of size *j* due to monomer addition, whilst the third and fourth terms relate to monomer dissociation. The final term in Equation (1) describes the creation of a growth nucleus consisting of *n_c_* monomers at a rate proportional to the monomer concentration raised to this power. In addition to the direct coalescence of *n_c_* monomers, pre-equilibrium and other schemes also result in the homogeneous nucleation term taking this form [14–25].

For the case of irreversible biofilament growth, the polymerisation rate dominates over the depolymerisation rate; from Equation (1), the rate of change of the number of filaments, *P*(*t*), and the free monomer concentration, *m*(*t*), were shown [1,8] under these conditions to obey:

(2) $$\frac{dP}{dt}=k_{n}m{\left(t\right)}^{n_{c}}$$

(3) $$\frac{dm}{dt}=-2k_{+}m(t)P(t)$$

Combining Equations (2) and (3) yields a differential equation for the free monomer concentration [1]:

(4) $$-\frac{d^{2}}{{dt}^{2}}\text{log}(m(t))=2k_{+}k_{n}m{\left(t\right)}^{n_{c}}$$

Here, we integrate these equations in the general case where the initial state of the system can consist of any proportion of monomeric and fibrillar material; this calculation generalises the results presented by Oosawa to include a finite concentration of seed material present at the start of the reaction. Beginning with Equations (2) and (3), the substitution *z*(*t*) := log(*m*(*t*)) followed by multiplication through by *dz/dt* yields:

(5) $$-\frac{d}{dt}\left[\frac{n_{c}}{4k_{+}k_{n}}{\left(\frac{dz}{dt}\right)}^{2}\right]=\frac{d}{dt}e^{n_{c}z}$$

Integrating both sides results in:

(6) $$-\frac{n_{c}}{2}{\left(\frac{dz}{dt}\right)}^{2}=2k_{+}k_{n}e^{n_{c}z}-A=-\frac{d^{2}z}{{dt}^{2}}-A$$

This is a separable equation for *dz/dt*, for which the solution is given by:

(7) $$\frac{dz}{dt}=\sqrt{\frac{2A}{n_{c}}}\text{tanh}\left(\frac{\sqrt{2{An}_{c}}}{2}(-t+2B)\right)$$

Integration and exponentiation yields the expression for *m*(*t*):

(8) $$m(t)={\left[\frac{A}{2k_{+}k_{n}}{\text{sech}}^{2}\left(\sqrt{\frac{{An}_{c}}{2}}(t-2B)\right)\right]}^{1/n_{c}}$$

The values of the constants *A* and *B* are fixed by inserting the appropriate boundary conditions in terms of *m*(0) and *P*(0). The mass concentration of polymers, *M*(*t*), is then provided through conservation of mass, *M*(*t*) = *m*_tot_ − *m*(*t*) with *m*_tot_ being the total monomer concentration. This results in the exact integrated rate law:

(9) $$M(t)=m_{\text{tot}}-m(0){\left[\mu \;\text{sech}\left(\nu +\lambda \beta^{-\frac{1}{2}}\mu t\right)\right]}^{\beta }$$

where the effective rate constant *λ* is given by $\lambda =\sqrt{2k_{n}k_{+}m{\left(0\right)}^{n_{c}}}$ and *β* = 2/*n_c_*, $\mu =\sqrt{1+\gamma^{2}}$, *ν* = arsinh (*γ*) for $\gamma =2k_{+}P(0)/(\beta^{\frac{1}{2}}\lambda )$.

We note that this expression only depends on two combinations of the microscopic rate constants, *k*_0_ = 2*k*_+_*P*(0) and *λ*. The result reveals that *λ* controls the aggregation resulting from the newly formed aggregates, whereas *k*_0_ defines growth from the pre-formed seed structures initially present in solution. In the special case of the aggregation reaction starting with purely soluble proteins, *P*(0) = 0, *m*(0) = *m*_tot_, these expressions reduce to *μ* → 1 and *ν* → 0, and Equation (9) yields the result presented by Oosawa [1] and the single relevant parameter in the rate equations is *λ*. Interestingly, generalisations of Equation (9) which include secondary pathways, maintain the dependence on *λ* and *k*_0_ but introduce an additional parameter analogous to *λ* for each active secondary pathway [26–29].

An expression for the evolution of the polymer number concentration, *P*(*t*) may be derived using Equation (9). Direct integration of Equation (2) gives the result for *P*(*t*):

(10) $$P(t)=P(0)+k_{n}m{\left(0\right)}^{n_{c}}\mu \frac{\text{tanh}(\nu +\beta^{-\frac{1}{2}}\lambda \mu t)-\text{tanh}(\nu )}{\beta^{-\frac{1}{2}}\lambda }$$

Equations (9) and (10) give in closed form the time evolution of the mass and number concentrations of biofilaments growing through primary nucleation and filament elongation.

### 2.2. Characteristic Features of Growth Involving Pre-Formed Seed Material

Insight into the early time behaviour of the polymer mass concentration can be obtained by expanding Equation (9) for early times to yield:

(11) $$M(t)\overset{t\to 0}{\to }M(0)+k_{0}m(0)t+m(0)[\lambda^{2}-k_{0}^{2}]t^{2}/2+O(t^{3})$$

This expression recovers the characteristic ~*t*^2^ dependence of the Oosawa theory and has an additional term linear in time relating to the growth of pre-formed aggregates.

In many cases, Equation (9) describes a sigmoidal function with a lag phase. The time of maximal growth rate, *t*_max_, can be found from the inflection point of the sigmoid from the condition *d*^2^*M/dt*^2^ = 0:

(12) $$t_{\text{max}}=\left[\text{artanh}\left(\sqrt{\frac{1}{1+\beta }}\right)-\text{arsinh }(\gamma )\right]{\left(\mu \beta^{-\frac{1}{2}}\lambda \right)}^{-1}$$

such that a lag phase exists only for:

(13) $$\text{artanh}\left(\sqrt{\frac{1}{1+\beta }}\right)>\text{arsinh }(\gamma )$$

Using the composition $\text{sinh}(\text{artanh}(x))=x/\sqrt{1-x^{2}}$ reduces this to the simple condition:

(14) $$k_{0}<\lambda$$

**Figure 1:**
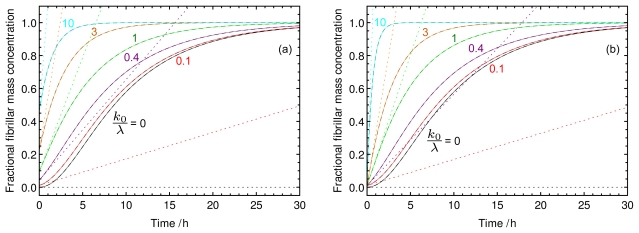
Nucleated polymerisation in the presence of seed material. The thick dashed lines are the exact solution to the rate equations Equation (9); the thin solid lines are calculated from numerical simulations of the master equation Equation (1). The dotted lines are the initial gradients *dM/dt*|*_t_*_=0_ = *M*(0) + 2*k*_+_*m*(0)*P*(0)*t*; a lag-phase exists when the initial gradient is not the maximal gradient. The numbers accompanying each curve are *k*_0_/*λ*; Equation (14) predicts that a lag-phase only exists when this ratio is less than unity. (**a**): Nucleated polymerisation in the presence of an increasing quantity of seed material of a fixed average length (5000 monomers per seed) added at the beginning of the reaction. The seed concentrations given as a fraction of the total concentration of monomer present are right to left): 0, 0.01, 0.04, 0.1, 0.2, 0.5; (**b**): Nucleated polymerisation in the presence of a fixed quantity (1% of total monomer in the system) of seed material of varying average length. The average number of monomer per seed are (right to left): N/A (unseeded), 5000, 1000, 500, 200, 50. The other parameters for both panels are: *m*_tot_ = 10 *μM*, *n_c_* = 3, *k_n_m* _tot_^*n*_*c*_−1^ = 1 · 10^−9^ s^−1^, *k*_+_ = 1 · 10^5^ M^−1^ s^−1^.

In other words, a point of inflection exists if the growth through elongation from the ends of pre-existing seeds, *k*_0_, is less effective that the proliferation through nucleation and elongation of new material *λ*. This result implies that an increased nucleation rate promotes the existence of an inflection point, whereas an increased elongation rate or an increased level of seeding tends to disfavour its existence. In particular, we also note that in the absence of nucleation, an inflection point cannot exist in the polymer mass concentration as a function of time. This result is illustrated in Figure 1 where kinetic profiles are shown for increasing levels of pre-formed seed material added at the beginning of the reaction; a lag-phase is only observed when *k*_0_/*λ <* 1, and the rate profile transforms from a sigmoidal shape at low pre-seeding levels to a concave form at high levels of seeding. Interestingly, the result Equation (14) is analogous to the criterion applicable for fragmentation dominated growth [26] where a lag phase exists only when the parameter controlling fragmentation-related secondary nucleation is larger than *ek*_0_.

The maximal growth rate, *r*_max_, is given by:

(15) $$r_{\text{max}}=\frac{2m(0)}{\sqrt{n_{c}(2+n_{c})}}{\left(\frac{2\mu^{2}}{2+n_{c}}\right)}^{\frac{1}{n_{c}}}\mu \beta^{-\frac{1}{2}}\lambda$$

which occurs at a polymer mass concentration *M*_max_ given from Equation:

(16) $$M(t_{\text{max}})=m_{\text{tot}}-m(0){\left(\frac{2\mu^{2}}{2+n_{c}}\right)}^{\frac{1}{n_{c}}}$$

The lag time, *τ*_lag_ := *t*_max_ − *M*(*t*_max_)/*r*_max_, is then given by:

(17) $$\tau_{\text{lag}}=\left[\text{artanh}\left(\sqrt{\frac{1}{1+\beta }}\right)-\text{arsinh }(\gamma )-\frac{m_{\text{tot}}-m(0){\left(\frac{2\mu^{2}}{2+n_{c}}\right)}^{\frac{1}{n_{c}}}}{\frac{2m(0)}{\sqrt{n_{c}(2+n_{c})}}{\left(\frac{2\mu^{2}}{2+n_{c}}\right)}^{\frac{1}{n_{c}}}}\right]{\left(\mu \beta^{-\frac{1}{2}}\lambda \right)}^{-1}$$

Interestingly, from Equation (17), we note that a point of inflection can never exist for *P*(*t*) for simple nucleated polymerisation. By contrast, when secondary pathways are active, an inflection point can frequently be present [27].

### 2.3. Comparison between Nucleated Polymerisation in the Presence and Absence of Secondary Pathways

Many systems that evolve through nucleated polymerisation display characteristic scaling behaviour, including power-law relationships between phenomenological parameters, such as the lag-time and maximal growth rate, and the initial concentration of monomeric peptide [26–31]. This behaviour can be seen to be a consequence of the fact that under many conditions, the rate equations are dominated by a single parameter that corresponds to the dominant form of nucleation: *λ* for classical nucleated polymerisation through primary nucleation, Equation (9), or an analogous parameter [21,26,29] *κ*_−_ or *κ*_2_, respectively, for polymerisation in the presence of filament fragmentation of monomer-dependent secondary pathways [11,21,26,29,32–34], Table 1. These parameters have the general form $\sqrt{2k_{+}m(0)k_{N}m{\left(0\right)}^{n}}$ where *k_N_* = *k_n_; k*_−_*; k*_2_ corresponds to the dominant nucleation process, *k_n_* for primary nucleation, *k*_−_ for filament fragmentation, *k*_2_ for monomer-dependent secondary nucleation, and *n* is related to the monomer dependence of this process: *n* = *n_c_* − 1, where *n_c_* is the critical nucleus size for primary nucleation, *n* = 0 for fragmentation driven growth and *n* = *n*_2_, the secondary nucleus size in cases where monomer-dependent secondary nucleation is dominant. The dominance of a single combination of the rate constants implies that many of the macroscopic system observables will be correlated since they are dependent on the same parameter. A striking examples of this behaviour is provided by the very general correlation between the lag-time and the maximal growth rate [29,30,35,36], which is manifested in the present case in Equations (15) and (17) as *r*_max_ ~ *λ* and *τ*_lag_ ~ *λ*^−1^.

Interestingly the rate equations describe sigmoidal curves both in the presence and in the absence of secondary nucleation processes. For more complex primary nucleation pathways [19,21] the polynomial form for the early time solution is maintained, but higher-order terms are obtained. This observation implies that in the absence of secondary processes the lag-phase is, in general, less marked since the early time rise is a slower polynomial relationship rather than the exponential onset characteristic of secondary pathways [21]. However, the difference between a high-order polynomial and an exponential may not be apparent in experimental data in the presence of noise, and therefore a global analysis of the system under different conditions is required in order to obtain robust mechanistic information. This might involve, for example [29], experimental measurements at varying initial concentrations of the monomeric peptide; a global analysis of the measured reaction profiles in terms of integrated rate laws and the related scaling behaviours can then be used to establish information about the microscopic mechanisms of proliferation [29].

## 3. Conclusions

In this paper, we have provided results for the time course of nucleated polymerisation for systems that are initially in a mixed state and contain both monomeric and fibrillar material. These results generalise the classical Oosawa theory that describes the formation of biofilaments to cases where an arbitrary amount of pre-formed seed material is present in the system. Furthermore, these results represent a reference to which polymerisation driven by secondary pathways can be compared.

## Figures and Tables

**Table 1:** Comparison of biofilament growth dominated by primary and secondary nucleation pathways. Primary nucleation processes create new aggregates at a rate that depends only on the concentration of monomeric peptide, whereas fragmentation creates new aggregates at a rate that depends only on the concentration of existing aggregates; monomer-dependent secondary nucleation creates new aggregates at a rate that depends on both the concentration of monomeric peptide and the concentration of existing aggregates. The dependencies of the latter two (secondary) nucleation processes on the existing aggregate concentration results in positive feedback: as the reaction proceeds, and proliferation through these mechanisms increases the concentration of aggregates, the rate at which these processes occur further is increased.

	Primary nucleation	Fragmentation	Monomer-dependent secondary nucleation
**Kinetic parameters**	*λ*, *k*_+_	*λ*, *κ*_−_, *k*_+_	*λ*, *κ*_2_, *k*_+_
**Early time growth**	Polynomial	Exponential	Exponential
**Scaling behaviour (lag time, max growth rate)**	Yes with *λ*	Yes with *κ*_−_	Yes with *κ*_2_
**Positive feedback**	No	Yes	Yes
